# Beyond the Earlobe: Navigating the Complexities of Relapsing Polychondritis

**DOI:** 10.7759/cureus.54293

**Published:** 2024-02-16

**Authors:** Sejoon Jun, Selina Chang, Amritpal Kooner, Bliss Colao, Evelyn Greco

**Affiliations:** 1 Dermatology, Walk-in Dermatology, New York City, USA; 2 Biomedical Engineering, Cornell University, Ithaca, USA; 3 Dermatology, University of Pittsburgh School of Medicine, Pittsburgh, USA; 4 Dermatology, Chicago College of Osteopathic Medicine, Downers Grove, USA; 5 Dermatology, University of Florida College of Medicine, Gainesville, USA; 6 Dermatology, New York Institute of Technology College of Osteopathic Medicine (NYITCOM), New York City, USA

**Keywords:** cartilage inflammation, chronic inflammation, autoimmune disorder, auricular chondritis, ear swelling, relapsing polychondritis clinical picture, polychondritis

## Abstract

A 27-year-old male with no significant past medical history presented with recurrent swelling and pain on the right superior crus of the antihelix initially misdiagnosed as a skin infection. Despite adherence to antibiotic treatment, his condition showed no improvement, leading to further investigation. The patient’s detailed clinical examination, family history devoid of autoimmune disorders, and persistent auricular inflammation prompted a reconsideration of the diagnosis. A subsequent biopsy that captured cartilage revealed auricular chondritis, perichondrial inflammation, degeneration of cartilage, and infiltration by inflammatory cells, all of which have been clinically associated with relapsing polychondritis (RP). Relapsing polychondritis (RP) is a rare autoimmune disorder characterized by recurrent inflammation of cartilaginous structures, often leading to progressive anatomical deformation and functional impairment. While RP's pathogenesis involves complex autoimmune mechanisms, its diagnosis is challenging due to its varied clinical presentations. This case highlights the diagnostic challenges of atypical presentations of RP and underscores the importance of considering RP in differential diagnoses of persistent auricular inflammation. It also emphasizes the role of corticosteroids in managing RP and the potential for novel therapeutic pathways, such as Janus kinase inhibitors, in treatment. The case contributes to a deeper understanding of RP's clinical spectrum and management strategies, stressing the need for heightened clinical suspicion in similar atypical cases.

## Introduction

Relapsing polychondritis (RP) is a rare autoimmune disorder characterized by recurrent inflammation of cartilaginous structures and connective tissues, notably the ears, nose, joints, and respiratory tract [1-3]. It has a variable incidence rate, with some studies suggesting a prevalence of approximately 3.5-4.5 cases per million people annually [1]. The exact pathogenesis of RP remains elusive, but it is believed to involve an autoimmune reaction against type II collagen in cartilaginous tissue [1-2,4]. Current hypotheses point towards the involvement of T-cell-mediated cytotoxicity and aberrant autoantibody production, leading to the degradation of cartilaginous tissue [4-5]. Various cytokines, including IL-1 and TNF-α, are thought to play a central role in the inflammatory cascade of RP, exacerbating the tissue damage. The Janus kinase (JAK) pathway, known for its cytokine signaling, has been hypothesized as a potential therapeutic target, given its involvement in similar autoimmune processes.

Management of RP is challenging due to its unpredictable course, potential for rapid progression, and involvement of critical anatomic structures [4,6]. Treatment strategies often involve corticosteroids and immunosuppressive agents; however, there is no universally effective therapy, and treatment is largely symptomatic and individualized. We present a case of a 27-year-old male with RP who was initially misdiagnosed as a simple skin infection, highlighting the diagnostic challenges and the need for heightened clinical suspicion in atypical presentations. The case underscores the complexity of RP management, the importance of early recognition, and the potential for exploring novel therapeutic pathways.

## Case presentation

**Figure 1:**
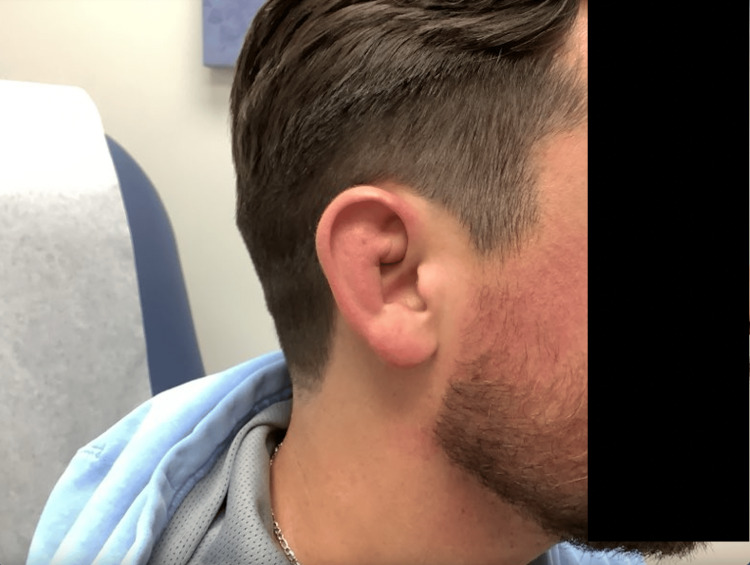
Clinical presentation of the patient showing continuous and confluent erythematous swelling of the superior crus of the antihelix of the right ear.

**Figure 2:**
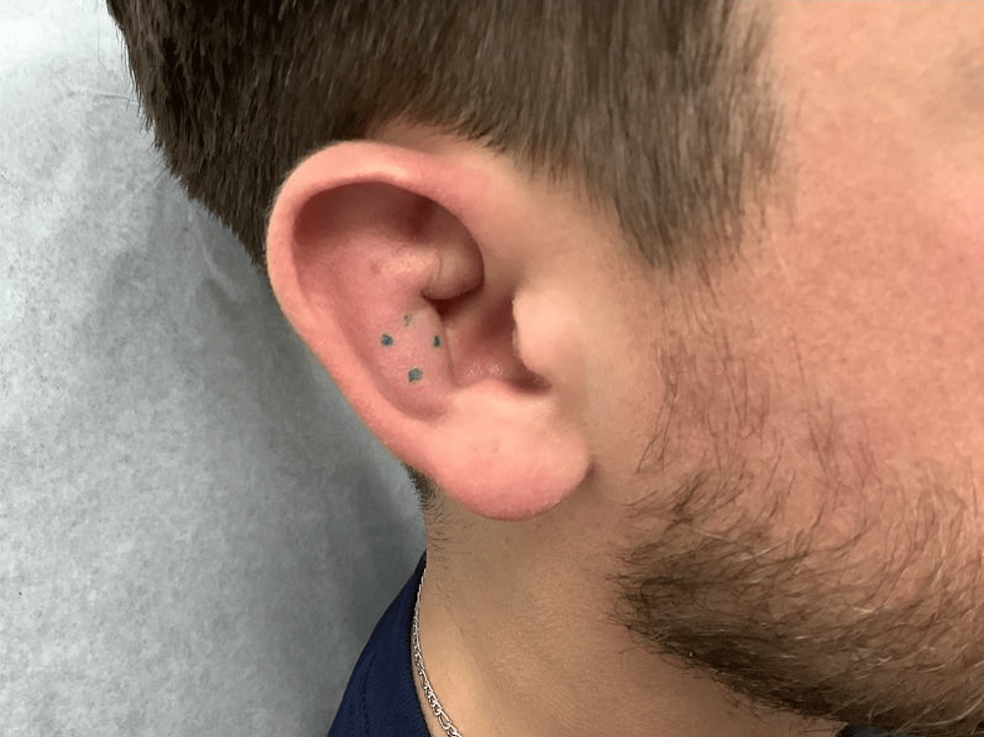
Photograph displaying a proximate view of the swelling of the right superior crus of the antihelix. Turquoise markings indicate the biopsy site.

A 27-year-old healthy male with no significant medical history was presented to our dermatology clinic for evaluation and treatment of a persistent skin irritation located on the right ear, initially diagnosed as a skin infection. His family history was notable for melanoma in his grandmother, and the patient reported no known family history of autoimmune disorders such as rheumatoid arthritis, systemic lupus erythematosus, or psoriasis. Additionally, he denied any cases of chronic inflammatory conditions like inflammatory bowel disease or multiple sclerosis. There was also an absence of constitutional symptoms such as patterns of recurrent fever, joint pains, and skin rashes that may have been suggestive of a hereditary predisposition to autoimmune or inflammatory diseases. Upon initial examination, the patient presented with swelling and pain in the superior crus of the right antihelix, with no history of trauma or recent illness. The patient disclosed that he predominantly sleeps on his right side, aligning with the unilaterally affected ear, and uses a firm pillow. He additionally mentioned that this swelling had been occurring recurrently, approximately every two weeks, for the past two months. Physical examination revealed bright erythematous, warm, and tender swelling of the auricular skin, with dermoscopic examination only showing erythema (Figures 1, 2). The patient had no involvement of the nose, and he denied any breathing difficulties or joint pain. There were no other rashes or skin lesions seen on his skin examination.

At the initial visit to our clinic, the patient was prescribed trimethoprim/sulfamethoxazole (800 mg-160mg tablet twice daily) for 10 days, and a bacterial culture was performed to rule out any infectious etiologies. Despite adherence to the antibiotic regimen, the patient’s condition showed no improvement at a follow-up visit one week later. The patient's persistent symptoms despite antibiotic treatment, combined with the localized inflammation of the auricular cartilage and a negative culture result, diverged from typical presentations of common skin infections. This atypical nature of the patient’s condition, along with a pattern of recurrence, prompted an expansion of our differential diagnosis to include relapsing polychondritis and a biopsy of the affected area was performed for further evaluation.

In the initial assessment, the biopsy results displayed *sparse perivascular dermatitis with telangiectases*, which were non-specific findings. However, upon a more detailed re-biopsy focused on the cartilage, significant pathological changes were identified. The deeper biopsy revealed auricular chondritis, perichondrial inflammation, marked cartilage degeneration, and infiltration by inflammatory cells.

As part of a comprehensive workup for persistent auricular inflammation, a series of investigations were conducted to evaluate for autoimmune connective-tissue diseases and vasculitis. The complete blood count (CBC) with differential revealed a slightly elevated white blood cell count of 11,500/µL (normal range: 4,500-11,000/µL), and a marginally low hemoglobin level of 11 g/dL (normal range for men: 13.8-17.2 g/dL). The platelet count was normal or slightly elevated at 350,000/µL (normal range: 150,000-450,000/µL). The metabolic panel showed electrolytes, blood urea nitrogen, and creatinine within normal limits, although serum creatinine was at the upper boundary of the normal range (1.2 mg/dL; normal range: 0.6-1.2 mg/dL). Liver function tests, including aspartate aminotransferase (AST, 25 U/L) and alanine aminotransferase (ALT, 30 U/L), as well as serum alkaline phosphatase (70 U/L), were all within normal limits. Urinalysis revealed trace or negative protein and was negative for blood. White and red blood cells were within normal ranges in the microscopic evaluation of the sediment. Cryoglobulins were noted but not quantified in routine tests. The viral hepatitis panel was negative for hepatitis A, B, and C. Autoimmune screening showed a low-titer positive antinuclear antibody (ANA) result. Antineutrophil cytoplasmic antibody (ANCA) was either negative or showed a non-specific pattern.

Given the lack of response to the initial treatment, we transitioned to a course of systemic corticosteroids. This decision was based on the established efficacy of corticosteroids in managing RP, particularly in controlling inflammation and preventing further cartilage damage. The specific regimen included prednisone, initiated at a dose of 40 mg per day. This dosage was selected to achieve rapid control of the inflammation, which is critical in managing acute episodes of RP.

The patient was advised to take prednisone in a single morning dose, as this helps to maintain an unaffected circadian rhythm. Additionally, we planned to taper the dosage gradually over a period of six weeks, monitoring the patient's response and adjusting the regimen as needed. The tapering strategy aimed to minimize the risk of potential long-term side effects of corticosteroids, such as osteoporosis, hypertension, and hyperglycemia.

After the six-week course, the patient responded remarkably well to the corticosteroid therapy, with complete resolution of swelling and pain in the affected ear. This positive outcome not only validated the diagnosis of RP but also highlighted the effectiveness of corticosteroids in the management of this condition. The successful resolution of symptoms with corticosteroid treatment underscores the importance of tailoring the therapeutic approach to the specific clinical scenario, especially in cases of atypical presentations.

## Discussion

This case offers a detailed insight into the challenges of diagnosing and managing relapsing polychondritis, a rare autoimmune disorder [4-9]. It is characterized by recurrent inflammation of cartilaginous structures, often leading to progressive anatomical deformation and functional impairment [4,7,10]. Apart from the recurrent unilateral auricular inflammation observed in our patient, RP can present in various forms, including auricular chondritis, nasal chondritis, ocular inflammation, respiratory tract involvement, joint symptoms, skin lesions, and audiovestibular damage [2]. These myriad presentations reflect RP's systemic nature, contributing to the challenges in its diagnosis and the potential for misdiagnosis in other conditions. The atypical presentation in our case, with the absence of some common features like bilateral involvement, further emphasizes the need for a comprehensive diagnostic approach.

The biopsy findings in our patient of auricular chondritis, perichondrial inflammation, and marked cartilage degeneration were significant. These pathological features, though not exclusive to RP, complemented the clinical picture and supported the diagnosis in a case where typical bilateral involvement was absent. This underscores the importance of considering RP in the differential diagnosis, even in cases with unusual presentations.

The laboratory workup in this case also plays a crucial role in understanding and managing RP. The slightly elevated white blood cell count and marginally low hemoglobin level might reflect the systemic inflammatory nature of RP, albeit non-specifically. Normal liver function tests and kidney parameters indicated the absence of systemic organ involvement, which can sometimes accompany autoimmune disorders [4,7,10]. The presence of trace protein in urinalysis, while not definitive, could suggest a mild systemic response. The low-titer positive antinuclear antibody (ANA) result, though not specific, is aligned with an autoimmune process. However, the absence of specific autoantibodies often seen in other connective tissue diseases has helped to narrow down the differential diagnosis [1-2,11].

Recent studies and clinical reports have advanced our understanding of RP, identifying various immunosuppressive and biological agents as potential treatment options [1-2,11]. Among these, corticosteroids remain the mainstay of therapy, as evidenced by their efficacy [11]. Other treatments, including methotrexate, TNF inhibitors, and biologics such as tocilizumab and rituximab, have shown promise in managing RP, but more robust data from controlled trials is needed for conclusive evidence of their efficacy [11,12].

The diagnostic journey in our case of relapsing polychondritis (RP) exemplifies the complexities inherent in distinguishing RP from other conditions with similar presentations. The differential diagnosis for atypical auricular inflammation includes a range of autoimmune, infectious, and structural disorders, each with unique characteristics [13]: 

Rheumatoid arthritis (RA): It may involve the auricular cartilage but typically presents with symmetrical joint involvement and is often associated with positive rheumatoid factor or anti-CCP antibodies, distinguishing it from RP.

Polyarteritis nodosa (PAN): It is characterized by systemic vasculitis and can be differentiated from RP through systemic symptoms, absence of cartilage-specific inflammation, and angiographic findings.

Cogan syndrome: This rare disorder presents with ocular and audiovestibular symptoms. Diagnosis relies on audiometric tests and ocular examination, which are distinct from RP.

Infectious perichondritis: It is caused primarily by bacterial infection, and it differs from RP in its acute onset, response to antibiotics, and culture results.

Mouth and genital ulcers with inflamed cartilage (MAGIC) syndrome: This involves symptoms of both RP and Behçet's disease. The presence of oral and genital ulcers, uveitis, and skin lesions helps in its identification.

Trauma: Particularly in sports like boxing or wrestling, trauma-induced auricular inflammation can be discerned from RP through clinical history and the absence of systemic symptoms.

Congenital syphilis: This presents with the Hutchinson triad and is confirmed through serological testing, differentiating it from RP.

Chronic external otitis: Characterized by inflammation of the ear canal, often secondary to infections or skin conditions, it can be identified through otoscopic examination.

Auricular calcification: Seen in conditions like Addison's disease, diabetes, or hyperthyroidism, it is diagnosed based on patient history and radiological findings. 

Our patient's remarkable response to corticosteroid therapy is a testament to the efficacy of this treatment modality in RP. Corticosteroids, a mainstay in RP management, not only alleviate symptoms but can also prevent further cartilage damage, as demonstrated in this case. The rapid improvement of our patient upon receiving prednisone, and the subsequent tapering strategy, highlights the critical role of corticosteroids in controlling acute flares of RP and points to the necessity of individualized treatment plans [1-2,11].

The complexity of RP's immunopathogenesis, involving cytokine dysregulation and T-cell-mediated responses, opens up possibilities for targeted therapies [1-2,5,10]. Janus kinase (JAK) inhibitors, which have shown effectiveness in other autoimmune conditions due to their role in cytokine signaling, might offer a new therapeutic pathway for RP [14]. However, their use in RP is still exploratory and would benefit from further research.

In light of this, the management of RP requires a multidisciplinary approach, considering the patient's symptoms, disease severity, response to initial therapies, and potential side effects [6,9,15]. The importance of early and accurate diagnosis cannot be overstated, as delayed treatment can lead to irreversible damage to cartilaginous structures [6,9]. 

This patient's case highlights the need for heightened clinical suspicion and a thorough investigation of cases of atypical auricular inflammation [6-9]. It underscores the importance of considering relapsing polychondritis in the differential diagnosis of persistent auricular inflammation and also brings attention to the need for developing long-term, sustainable treatment strategies in RP.

## Conclusions

Our case underscores the complexity of diagnosing and treating relapsing polychondritis (RP), a condition marked by its unpredictable progression and diverse clinical presentations. The journey of our patient from initial misdiagnosis to successful management illustrates the necessity of continuous advancements in our understanding of RP's pathogenesis. It emphasizes the critical role of ongoing research, particularly in the development of targeted therapies, which hold the promise of significantly improving patient outcomes. This case serves as a reminder to the medical community of the imperative to remain vigilant in identifying RP, especially in atypical cases, and to be adaptive in embracing innovative therapeutic strategies. Our experience reinforces the call for a proactive and multidisciplinary approach to the management of RP, ensuring the highest standard of care for those affected by this challenging autoimmune disorder.
